# Rapid Plant Identification Using Species- and Group-Specific Primers Targeting Chloroplast DNA

**DOI:** 10.1371/journal.pone.0029473

**Published:** 2012-01-12

**Authors:** Corinna Wallinger, Anita Juen, Karin Staudacher, Nikolaus Schallhart, Evi Mitterrutzner, Eva-Maria Steiner, Bettina Thalinger, Michael Traugott

**Affiliations:** Mountain Agriculture Research Unit, Institute of Ecology, University of Innsbruck, Innsbruck, Austria; University of Minnesota, United States of America

## Abstract

Plant identification is challenging when no morphologically assignable parts are available. There is a lack of broadly applicable methods for identifying plants in this situation, for example when roots grow in mixture and for decayed or semi-digested plant material. These difficulties have also impeded the progress made in ecological disciplines such as soil- and trophic ecology. Here, a PCR-based approach is presented which allows identifying a variety of plant taxa commonly occurring in Central European agricultural land. Based on the *trn*T-F cpDNA region, PCR assays were developed to identify two plant families (Poaceae and Apiaceae), the genera *Trifolium* and *Plantago*, and nine plant species: *Achillea millefolium*, *Fagopyrum esculentum*, *Lolium perenne*, *Lupinus angustifolius*, *Phaseolus coccineus*, *Sinapis alba*, *Taraxacum officinale*, *Triticum aestivum*, and *Zea mays.* These assays allowed identification of plants based on size-specific amplicons ranging from 116 bp to 381 bp. Their specificity and sensitivity was consistently high, enabling the detection of small amounts of plant DNA, for example, in decaying plant material and in the intestine or faeces of herbivores. To increase the efficacy of identifying plant species from large number of samples, specific primers were combined in multiplex PCRs, allowing screening for multiple species within a single reaction. The molecular assays outlined here will be applicable manifold, such as for root- and leaf litter identification, botanical trace evidence, and the analysis of herbivory.

## Introduction

The identification of plants is well established for the majority of species occurring in Europe. This approach, however, gets corrupted when no morphologically assignable parts are available [1], [2]. For example, determining the composition of root samples containing multiple species by morphology-based approaches is impossible [3]. The problem becomes even more evident when bringing soil-living herbivores into play. Albeit leaf litter and below-ground plant parts are representing an important food source, our knowledge of the dietary choice in soil-living animals is rudimentary [4]. The semi-digested plant tissues, remaining in the intestine or faeces of herbivores, are also not identifiable based on morphological characters. Knowing the food sources and dietary preferences of soil animals, however, is vital, for example to manage soil insect pests.

Molecular methods, based on genomic differences between plant species, offer a promising means to circumvent these problems [5], [6]. In recent years, the application of diverse molecular techniques has gained increasing importance in answering ecological questions, e.g. concerning population genetics, the assessment of invasive or endangered species, or trophic interactions based on morphologically unidentifiable remains [7]. Amongst these newly evolved approaches, DNA barcoding, which relies on the use of a standardized DNA region for accurate and rapid species identification [8], has been used more and more by ecologists. Since the last decade the international initiative CBOL (Consortium for the Barcode of Life, http://barcoding.si.edu) aims in global standards for DNA barcoding. But, in plants the situation is controversial and many strategies have been proposed. The mitochondrial cytochrome *c* oxidase subunit I gene (COI), which serves as the standard barcode for animals, is not suitable for species identification in plants, due to low levels of variability. Previous studies on DNA-based plant identification were primarily focusing on the plastid genome (e.g. [9], [10], [11], [12], [13]), but there is a lack of consensus regarding the most universal, informative and technically practical DNA region(s). The suitability of a molecular marker strongly relies on the questions to be answered. For ecologists, who are concerned with the identification of environmental samples [7] it is essential, that the target DNA region exhibits highly conserved priming sites to guarantee reliable DNA amplifications. Moreover, it should be short enough to allow amplification of degraded DNA. Taberlet et al. [14] promoted the *trn*L intron as a plant barcode, harbouring its main power in ecological applications [7], i.e. when working with degraded DNA [15], [16], [17], [18].

The *trn*L-barcode has also been adopted in studies on herbivory using next generation sequencing techniques [19], [20], [21], [22]. This approach, however, is costly, especially for processing large numbers of individual samples. Diagnostic PCR (polymerase chain reaction) using specific primers, offers a cost-effective alternative for the molecular identification of specific plant taxa. If the primers are designed to amplify amplicons of different length, it is possible to screen for multiple species within a single reaction [23], [24]. However, unlike the COI in animals, the alignment of non-coding cpDNA sequences is challenging due to the considerable variability which can occur even between closely related taxa [25]. Consequently, so far only few primers are available for specific plant taxa, most of them accessing nuclear DNA [1], [26], [27]. The current paper describes a novel approach for identifying plant species via diagnostic PCR based on the *trn*L-F region.

Here we present diagnostic PCR assays, ready to use for the identification of various plant taxa common in agricultural land. Moreover, we show - step by step - how to generate these PCR assays using primers targeting the *trn*T-F cpDNA region, allowing the development of diagnostic assays for further plant taxa not included here. Our approach involves three consecutive steps: (i) development of specific plant primers at different taxonomic levels, (ii) combination of primers in multiplex reactions, and (iii) optimization of PCR protocols to maximize their specificity and sensitivity. This practice involves two deliberate strategies, aiming to maximize screening efficacy: firstly, the development of group-specific primers allows a pre-selection, thus reducing the number of samples that need to be analysed for different species within the respective genera or families (2-step analysis); and secondly, the combination of primer pairs in multiplexes to reduce the number of PCRs necessary.

## Methods

The current paper is part of a comprehensive study on the feeding ecology of wireworms, the soil-living larvae of click beetles (Coleoptera: Elateridae). Wireworms of the genus *Agriotes* were chosen as they feed on the underground parts of a wide range of plants [28] and are amongst the most abundant soil pests in arable land [29]. The locations of plant and animal collection were not protected in any way and no specific permits were required for the described studies. We confirm that they did not involve endangered or protected species.

To identify the plant species eaten by these insect larvae we employed a PCR- approach, based on the use of specific primers. Each primer combination was designed to specifically target a single plant species, genus or family, thus resulting in a DNA fragment of distinctive size allowing identifying the targeted taxa. The best performing primer combinations were then joined together in multiplex PCRs, and reaction conditions optimized for maximum specificity and sensitivity.

To develop and test the method there had been five consecutive steps: (i) compilation of a sequence database, (ii) construction of specific primers based on the sequence database, (iii) test of primers and optimization of PCR reactions, (iv) evaluation of the developed PCR-assays for specificity and sensitivity, (v) test of the PCR-assay on various field samples.

### Plant species and DNA extraction

All plants (target and non-target species, Table 1) were collected as multiple individuals in the summers 2008/09 from grasslands and maize fields in Tyrol (Austria) and stored at −80°C.

**Table 1:** Plant species collected in maize fields (M) and perennial grassland (G), which were used to establish the PCR-based identification system.

Plant Species	M	G	Plant Species	M	G
***Achillea millefolium***	✗	✗	*Lotus corniculatus*		✗
***Aegopodium podagraria***	✗	✗	***Lupinus angustifolius***	✗	
*Ajuga reptans*	✗	✗	*Medicago lupulina*		✗
*Alchemilla vulgaris*		✗	*Medicago sativa*		✗
***Anthoxanthum odoratum***		✗	*Melilotus albus*	✗	
***Anthriscus sylvestris***		✗	*Melilotus officinale*	✗	
***Arrhenatherum elatius***		✗	*Myosotis arvensis*		✗
***Avena sativa***		✗	*Papaver rhoeas*		✗
***Avenula pubescens***		✗	*Persicaria maculata*	✗	✗
*Bellis perennis*	✗	✗	***Phaseolus coccineus***	✗	
*Beta vulgaris*	✗		***Phleum pratense***	✗	✗
*Brassica napus*		✗	***Pimpinella major***		✗
*Brassica nigra*		✗	***Plantago lanceolata***	✗	✗
*Brassica oleracea*	✗		***Plantago major***		✗
***Bromus hordeaceus***		✗	***Poa pratensis***	✗	✗
*Campanula patula*		✗	***Poa trivialis***		✗
*Capsella bursa-pastoris*	✗	✗	*Polygonum aviculare*		✗
*Cardamine pratensis*		✗	*Prunella vulgaris*		✗
***Carum carvi***	✗	✗	*Ranunculus acris*	✗	✗
*Centaurea jacea*		✗	*Ranunculus ficaria*		✗
*Centaurea scabiosa*		✗	*Ranunculus repens*	✗	✗
*Cerastium holosteoides*	✗	✗	*Rumex acetosa*		✗
*Chenopodium album*	✗	✗	*Rumex crispus*	✗	✗
*Chenopodium polyspermum*	✗		*Salvia pratense*		✗
*Cichorium intybus*	✗		*Senecio vulgaris*	✗	
*Cirsium arvense*	✗	✗	***Setaria viridis***	✗	
*Convolvolus arvensis*		✗	*Silene dioica*	✗	✗
***Dactylis glomerata***	✗	✗	*Silene latifolia*	✗	✗
***Digitaria ischaemum***	✗		*Silene vulgaris*		✗
***Echinochloa crus-gallii***		✗	***Sinapis alba***	✗	
*Euphorbia helioscopia*		✗	*Solanum nigrum*		✗
***Fagopyrum esculentum***	✗		*Sonchus asper*	✗	
***Festuca pratensis***	✗	✗	*Sonchus oleraceus*	✗	✗
*Galeopsis tetrahit*		✗	*Stellaria media*		✗
*Galinsoga ciliata*	✗	✗	*Symphytum officinale*		✗
*Galium mollugo*	✗	✗	***Taraxacum officinale***	✗	✗
*Geranium robertianum*	✗		*Tragopogon pratense*		✗
*Glechoma hederacea*	✗	✗	***Trifolium pratense***	✗	✗
*Helianthus annus*		✗	***Trifolium repens***	✗	✗
***Heracleum sphondylium***		✗	***Trisetum flavescens***		✗
*Hieracium pilosella*	✗		***Triticum aestivum***	✗	
***Holcus lanatus***		✗	*Urtica dioica*	✗	
*Knautia arvensis*		✗	*Veronica arvensis*		✗
*Lactuca seriola*	✗		*Veronica chamaedrys*		✗
*Lamium purpureum*		✗	*Veronica filiformis*		✗
*Lathyrus pratensis*		✗	*Vicia cracca*	✗	
*Leontodon hispidus*		✗	*Vicia faba*	✗	
*Leucanthemum ircutianum*		✗	*Vicia sativa*		✗
***Lolium multiflorum***		✗	*Vicia sepium*		✗
***Lolium perenne***	✗	✗	***Zea mays***	✗	

Target species of the molecular assays are displayed in bold.

Plant tissue was homogenized together with glass beads in 440 µL lysis buffer containing TES-buffer (0.1 M TRIS, 10 mM EDTA, 2% SDS, pH 8), 10 µL Proteinase K (20 mg/mL, AppliChem, Darmstadt, Germany), and a pinch of PVP (Polyvinylpyrrolidone) using a Precellys® 24 Tissue Homogenizer (Bertin Technologies, Montigny-le-Bretonneux, France). To increase the DNA yield, samples were incubated in the lysis buffer for 12 hours. The remaining DNA extraction followed a modified CTAB-based protocol described by Juen & Traugott (2005). Forty seven plant species were sequenced for part or the whole cpDNA sequence of interest, and representative sequences submitted to GenBank (accession numbers are JQ041821 – JQ041881).

### Sequence Database

The chloroplast DNA sequence between the *trn*T (UGU) and the *trn*F (GAA) genes was selected for the development of the species-, genus- and family-specific primers. This region comprises two exons of the *trn*L (UAA) gene (*trn*L-E1 and *trn*L-E2) and three non-coding regions: the intergenic spacer between *trn*T and *trn*L-E1 (IS1), the *trn*L intron (*trn*L-I) and the intergenic spacer between *trn*L-E2 and *trn*F (IS2). This chloroplast region is known for its potential as species-specific marker due to low intra- and higher inter-specific genetic variation [14]. Primer design was based on alignment of sequences from target and non-target plant species. The sequence database was built by combining published sequences from GenBank and sequencing results from specimens collected in grasslands and maize fields at the study sites. Of the 100 plant species present (Table 1) 78 species were represented by part or the whole cpDNA sequence of interest in GenBank already. Using general primers [14], [10], [15] (PCR conditions see: Supporting Information S1) we obtained sequences of additionally 46 species (GenBank accession numbers are JQ041821 – JQ041881). Altogether we relied on a final sequence database comprising 92 plants.

Since the entire *trn*T-F region is too long for sequencing it within a single sequence run, several reactions need to be carried out, resulting in a final assemblage of the entire region. But, the general plant primers [14] do not always perfectly match, resulting in incomplete DNA sequences for some species, both in our sequence database and in GenBank.

Sequence information on the introns *trn*L-I and the intergenic spacer IS2 was available for 91% and 80% of the investigated plants, respectively. Fewer sequences could be retrieved for the IS1 (36% of the investigated plant species. Sequences length varied from 241 to 588 bp for the *trn*L-I, and 541 to 991 bp and 75 to 692 bp for the intergenic spacers IS1 and IS2, respectively. Consensus sequences for each species were constructed by combining all sequence information available using BioEdit Sequence Alignment Editor [30].

### Primer design

An overall reliable sequence alignment of all study species was impossible due to the high variability within the non-coding regions and the fact that for many of the species only part of the *trn*T-F cpDNA was available. So we aligned (i) all sequences within families and (ii) all sequences that were available in full length, i.e. the whole sequence between *trn*T and *trn*F (30 plant species) using Clustal X (Larkin et al. 2007). Finally, the alignments were hand-edited using BioEdit. Based on these sequence alignments it was possible to define regions that were highly similar across all species and families and we could pinpoint sequence positions that were suitable for the 3′-end of the specific primers.

Forward and reverse primers were constructed for different plant taxa using CLC DNA Workbench 4.0, (CLC bio, Aarhus, Denmark) following the rules for ARMS primer design (Hawkins 1997). We developed group- and species-specific primers to identify two plant families (Poaceae and Apiaceae), the genera *Plantago* and *Trifolium*, and nine plant species common in Central European agricultural land: *Achillea millefolium*, *Fagopyrum esculentum*, *Lolium perenne*, *Lupinus angustifolius*, *Phaseolus coccineus*, *Sinapis alba*, *Taraxacum officinale*, *Triticum aestivum*, and *Zea mays.* All potential primers were checked in CLC DNA Workbench for cross-amplification, within target and non-target species. Only 10% of the originally selected primer positions were found reliable for specific primers, due to repeats of sequences on both strands and in different relative positions within introns. The evaluation of the primers included tests of several DNA extracts from at least five different individuals per plant species. The final primer pairs were chosen based on similarity in melting temperature and on the fragment length of amplicons.

### Optimization of PCR assays

All primers developed were initially checked in singleplex PCRs (specific conditions see: Supporting Information S2). The best performing primer combinations were then tested in gradient PCRs to define the optimum annealing temperature. Finally, conditions for multiplex PCRs were optimized, testing different concentrations of primers (0.2–0.8 µM) and MgCl_2_ (3–6 mM), and by varying the duration of annealing and extension steps (60 or 90 s). To test the efficiency of the assays in amplifying specific taxa in compound samples, mixes from the targeted plant DNA in different combinations were used. The mixed samples included DNA of different numbers and combinations of target and non-target species. PCR products were visualized on QIAxcel, an automated capillary electrophoresis system (Qiagen, Hilden, Germany) with method AL320, and results were scored using BioCalculator Fast Analysis Software version 3.0 (Qiagen). All samples showing the expected fragment length, with signal strength above 0.1 relative fluorescent units, were deemed to be positive.

### Evaluation of the PCR assays

The specificity of the primer pairs finally selected was tested for cross-amplification against DNA from all other species occurring in the same habitat (i.e. grasslands and maize fields; Table 1) and against wireworm DNA.

For testing the sensitivity of the newly established PCR assays, DNA templates of all target species for species-specific primers and of representative species for the genus- and family-specific primers were required (Table 2). Hence, general plant primers [14] were used to amplify fragments from the *trn*T-F cpDNA region which covered the binding sites of the newly designed primers (PCR conditions are given in Supporting Information S3). The DNA concentrations of the purified PCR products were then determined with a VICTOR™×4 Multilabel Plate Reader (Perkin Elmer, Waltham, USA) using Quant-iT™ PicoGreen® dsDNA Assay Kit (Invitrogen, Paisley, UK) and the molecular weight of the PCR products was computed, summarizing the weight of the nucleotides within the sequences of each species (including the flanking primer sequences). Based on the DNA concentrations (ng µL^−1^) and the molecular weight of the fragments the number of template copies per ng DNA was calculated, which was finally used for sensitivity testing.

**Table 2:** Details of primers: plant species targeted, primer sequences (forward- followed by reverse primer), expected amplicon length, concentration of each primer (µM), optimized annealing temperatures (°C), MgCl_2_ concentration (mM), and affiliation to a multiplex assay.

Target taxa	Primer name	Primer sequence (5′-3′)	Size (bp)	Conc. (µM)	°C	MgCl_2_(mM)	Multiplex assay
*Fagopyrum esculentum*	Fag-sp-S519	gaaaacgaaaggaaaggttcat	380	0.2	56	4	FLPS
	Fag-sp-A523	caggattacccgttttttga					
*Lolium perenne*	Lol-per-S528	gcatttttctatatagaatggat	254	0.4			
	Lol-per-A535	tgactctatgttctccttagtt					
*Phaseolus coccineus*	Pha-sp-S525	atcctttcacaaaaattccag	235	0.2			
	Pha-sp-A531	tggatcagttcttcaagggt					
*Sinapis alba*	Sin-alb-S534	attcactaaactactagatcgt	203	0.4			
	Sin-alb-A542	catgaaatcaaaattcgaaagtc					
*Lupinus angustifolius*	Lup-sp-S522	gaatccattcaacagttctg	244	0.2	53	4	LF
	Lup-sp-A527	gaacttttctttgtttttgcg					
*Fagopyrum esculentum*	Fag-sp-S519	gaaaacgaaaggaaaggttcat	206	0.2			
	Fag-sp-A524	tattaccctttcataccgcat					
*Taraxacum officinale*	Tar-sp-S546	cggttcaaaactcctttatg	194	0.2	58	4	TAT
	Tar-sp-A554	ttcctcatgtctcatcctt					
*Achillea millefolium*	Ach-sp-S547	gcggttcaaaattccttatac	222	0.2			
	Ach-sp-A556	agggtattacaaagactcg					
*Trifolium repens*	Tri-sp-S550	cagtaggaaaggaatcgttct	172	0.2			
	Tri-sp-A558	aatctttcatttgtgatagaaaag					
*Trifolium pratense*	Tri-sp-S550	cagtaggaaaggaatcgttct	151	0.2			
	Tri-sp-A558	aatctttcatttgtgatagaaaag					
*Triticum aestivum*	Tri-aes-S536	gctattaactagttctaaatttgaagtta	306	0.5	54	4	TZ
	Tri-aes-A545	cctcccgtcttacttttttat					
*Zea mays*	Zea-may-S510	atttgatcattatatacatttttgagat	181	0.2			
	Zea-may-A539	tccttccttttttagagtattcc					
*Plantago* spp.	Pla-sp-S557	atctattttctagctatcctacc	116	0.5	61.5	4	
	Pla-sp-A565	cgcatgtgataagagaaagtc					
Apiaceae	Api-gen-S561	aatgaccgtctttgaccaaa	198/199	0.5	62	3	
	Api-gen-A569	attctcattcccgatatcgc					
Poaceae	Poa-gen-S541	gctttctcattctactctttc	187–293	0.2	56	3	
	Poa-gen-A551	cttttcttgtgcatcatcctag					

The genus- and family-specific primers were run in singleplex reactions.

The actual sensitivity of the optimized diagnostic PCR protocols was determined via serial dilution of template DNA (i.e. known numbers of copies). Assay sensitivity was also evaluated in the presence of wireworm DNA to test the capability for molecular gut content analysis. For the latter, for each plant species 1 µl of the two highest dilutions of template DNA tested positive (Table 3) were spiked with 3.5 µl of undiluted *Agriotes* spp. DNA.

**Table 3:** Plant species list used for determining sensitivity of multiplex (FLPS, LF, TAT, TZ) and singleplex assays (Plantago, Apiaceae, Poaceae).

Plant species	Detection limits of plant DNA		Assay type
	Plant DNA only	plus wireworm DNA	
*Fagopyrum esculentum*	200	200	FLPS
*Lolium perenne*	100	200	
*Phaseolus coccineus*	200	400	
*Sinapis alba*	100	200	
*Lupinus angustifolius*	100	200	LF
*Fagopyrum esculentum*	400	800	
*Taraxacum officinale*	100	100	TAT
*Achillea millefolium*	200	400	
*Trifolium pratense*	100	100	
*Trifolium repens*	100	200	
*Triticum aestivum*	100	200	TZ
*Zea mays*	100	200	
*Plantago lanceolata*	100	100	*Plantago* spp.
*Plantago lanceolata*	100	100	
*Anthriscus sylvestris*	800	800	Apiaceae
*Carum carvi*	100	100	
*Heracleum sphondylium*	100	200	
*Pimpinella major*	1,600	1,600	
*Avena sativa*	100	200	Poaceae
*Bromus hordeaceus*	100	200	
*Dactylis glomerata*	100	200	
*Digitaria ischaemum*	100	200	
*Lolium perenne*	100	100	
*Setaria viridis*	100	100	
*Trisetum flavescens*	100	100	

Lowest detection rates achieved are given in number of template copies.

### Applicability of the PCR assays

To evaluate the performance of the method with degraded and complex samples, DNA extracts of decayed plant material and wireworms from both, feeding experiments and catches in the field, were tested.

For decayed samples, maize stalks and whole wheat plants were buried in an abandoned field (574 m a.s.l., Tyrol, Austria) and left there for 20 (wheat) and 24 (maize) weeks, respectively. At this time point most plant parts were almost decomposed. We then analyzed ten DNA extracts per plant species using the TZ duplex (Table 2) to test the applicability of our method for decayed plant tissues.

In addition, we tested the PCR assays on whole-body extracts of wireworms obtained from feeding experiments, which were performed similar to those described in [31]: we offered *L. perenne*, *T. officinale*, *A. millefolium*, *T. pratense*, *Plantago lanceolata*, and *Pimpinella major* for 24 h to the larvae as a food source. Subsequently, total DNA of 10 wireworms per plant species was extracted, including any plant DNA present within their guts [31], and analyzed them with the adequate PCR assays (Table 3).

The third set of samples comprised whole-body DNA extracts of wireworms, which were collected in a maize field (574 m a.s.l., Tyrol, Austria); these samples were tested with the TZ duplex PCR (Table 2).

## Results

### Specific primers

**Figure 1:**
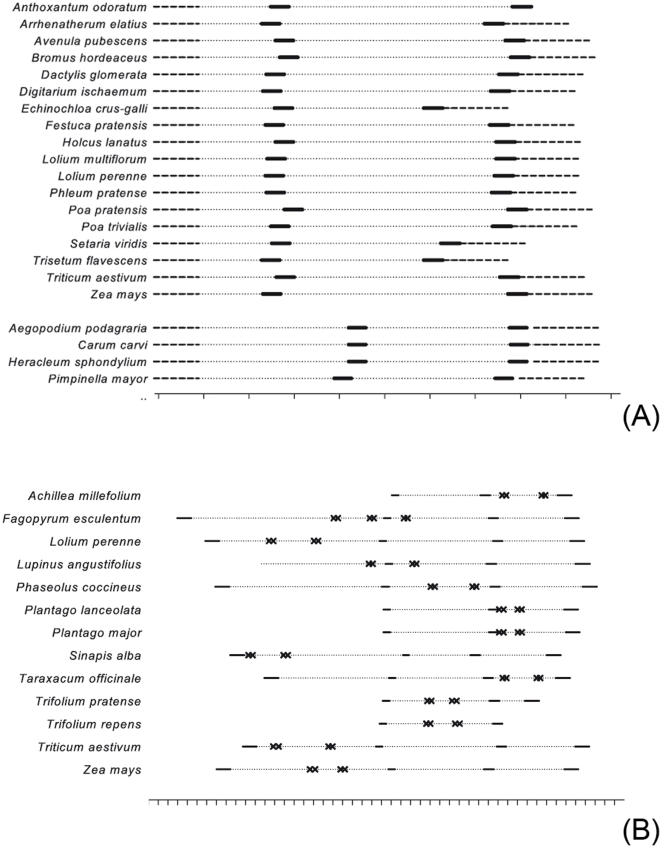
Relative location of primer binding sites on the *trn*T-F cpDNA region. At the base of each figure is a size marker, which indicates a sequence length of 50 bp. (**A**) Positions of the family specific primers for Poaceae (above) and Apiaceae (below): The dotted lines represent the known sequence, the dashed lines the second exon of *trn*L and the exon of *trn*F, and the thick bars symbolise the primer binding sites. (**B**) Position of the genus- and species specific primers: The dotted lines represent the known sequence, the inner bars indicate the position of the two *trn*L exons and the outer bars the position of the *trn*T and the *trn*F gen. The binding sites of primers are indicated by double crosses.

Species and genus-specific primer sites were found in all introns (Fig. 1), and the PCR products of two species-specific primer pairs also include the *trn*L-E1 region. The newly designed primers generate amplicons ranging between 116 bp and 381 bp.

The two family-specific primer pairs for Poaceae and Apiaceae are positioned in the IS2 and both reverse primers are placed next to the *trn*F gene. The length of the PCR product for Poaceae varies considerably among species, being shortest for *Echinochloa crus-galli* (187 bp) and longest for *Z. mays* (293 bp). In contrast, the amplicon length for Apiaceae is the same for all five species tested (198 bp). Likewise, the primers for the genus *Plantago* result in PCR products of the same length for the two species tested *(P. major* and *P. lanceolata*, 116 bp). The multiplex TAT, on the other hand, allows discerning between four different species within a single PCR (Table 3), because *T. repens* and *T. pratense*, were represented by different amplicon length (172 bp and 151 bp, respectively), using the very same primers (Tri-sp-S550 and Tri-sp-A558). For *F. esculentum* two primer combinations were optimized, resulting in fragments of 380 bp and 206 bp length, respectively. Assays for the remaining species-specific primers generate amplicons ranging between 181 (*Z. mays*) and 306 bp (*T. aestivum*) in length.

### Diagnostic PCR assays

Each PCR contains 4 µL of DNA extract per 15 µL reactions, 7.5 µL 2× TypeIt Mutation™ Detect PCR Kit (Qiagen), 0.5 µg bovine serum albumin (BSA), and 0.5 µL 5× Q-solution (Qiagen). The thermocycling program is: 95°C for 5 min, 40 cycles of 92°C for 20 s, 51–64°C for 90 s and 70°C for 90 s and finally 70°C for 5 min. Primer concentrations, MgCl_2_ content and annealing temperature for specific PCRs are given in Table 2.

In only one case non-target species generated PCR products which were of similar size than the ones of the targeted plants: the multiplex designed for *T. officinale*, *A. millefolium* and the two *Trifolium* species (TAT) cross-reacted with DNA of *Medicago lupulina*, producing a 222 bp fragment, the same length as the one expected for *A. millefolium*.

The PCR assays are highly sensitive: in most cases amplification and visualization of the target DNA is possible down to the presence of 100 templates of target DNA per PCR (Table 3). The presence of wireworm DNA does not or only marginally decrease the sensitivity of the different assays (Table 3). Only two Apiaceae species exhibit a lower sensitivity: *Anthriscus sylvestris* at 800 templates and *Pimpinella major* at 1,600 templates. For *F. esculentum* the primers amplifying the longer fragment turned out to be more sensitive (200 copies) than the ones generating the shorter one (400 copies).

### Applicability of the newly established PCR assays

In the decay experiment, all DNA extracts of the decayed parts from maize and wheat, that were recovered after 20 or 24 days exposure in the soil, could be identified (detection rate = 100%). Likewise, all plant species fed to the wireworms were detectable in the whole-body DNA extracts of larvae (the mean detection rate over all plant species was 30%). The detection rates were 50% for *P. major*, 45.5% for *P. lanceolata*, 29.2% for *A. millefolium*, 20% for *L. perenne*, 18.6% for *T. officinale*, and 10% for *T. pratense*. Out of the field-collected wireworms, 21% tested positive for maize DNA.

## Discussion

We present optimized PCR assays based on specific primers for the identification of plant DNA. Based on a discrimination of similar *vs*. variable sequence regions within and among families and a comprehensive testing of cross-reactivity of primers *in silico* we were able to generate specific primers targeting the *trn*L-F cpDNA region. The most challenging part within the development of these assays was the development of reliable primers. This is mainly due to the highly ambiguous alignment of the selected chloroplast sequences caused by high rates of indels – a general feature of cpDNA spacer regions [25]. It appears that even within closely related taxa, great length differences in non-coding regions exist, such that at greater taxonomic distances no shared sequences remain.

Earlier attempts of molecular identification from morphologically indistinguishable plant parts employed different DNA markers and methods. Some of the methodological hurdles involved are coinciding with the difficulty in finding an appropriate DNA barcode for plants. The ITS, for example has been successfully applied to distinguish plants in small scale studies harbouring a limited number of species [32], [1], [33]. But it does not always allow to identify plant species unambiguously [6]. Likewise, Kesanakurti et al. [34] were unable to distinguish multiple species using the *rbc*L for the identification of plant roots. Consequently, more rapidly evolving regions are required when barcoding roots, or the application of a 2-locus approach, as promoted by the CBOL plant working group [35]. In addition, alternative PCR-based methods have been applied [36], [37], [16]: DNA sequencing or restriction fragment length polymorphism (RFLP) analysis of plastid genes (*rbc*L and *trn*L). Moore and Field [32] were able to identify root samples of up to four species based on RFLP keys. Despite their usability, RFLPs reveal only changes at restriction sites or length variation large enough to be detected [6]. With an increasing number of species present in a sample the revealed patterns are more likely to blend together and overlap. Moreover, the type of the organ, where the DNA is taken from, affects the genetic fingerprint, as pattern differences between roots and leaves were found [38].

Each of the approaches described above comprises a cascade of reactions necessary to assign PCR products to a specific plant species. Contrary, we could identify plants to species level within a single PCR. The *trn*T-F cpDNA used in this study already proved as an appropriate barcode for identifying digested plant DNA [39], [40], [41]. But, the approach presented here is also applicable for other loci than the *trn*T-F region. Once specific primers are established, multiplex PCR provides a means to detect and identify several targets simultaneously [42]–[43], circumventing the need of follow-up reactions such as RFLP analysis. The number of species that can be identified simultaneously in a single multiplex PCR is limited due to the requirement of adequate size differences between the amplicons, and in case degraded plant DNA is targeted, by the restricted length of the PCR products [23]. As the number of target species increases, so will the time and effort needed to screen each sample for multiple plant species. Another limitation of our approach is the need to sequence and find primer sites prior to the application of a new PCR system. In time, an increasing number of both, plant sequences and specific primers will become available, thus reducing these efforts. This process could also be accelerated by the use of next-generation sequencing, which is capable of sequencing many thousands of samples simultaneously [44].

Whereas with our approach only plant taxa are accessible for which primers already are developed, next-generation sequencing allows an examination without a priori knowledge of the species involved. But, this approach also implies that the general primer used, match equally well on all target species and that preferential amplification of certain species does not inhibit the detection of other species [45]. Besides, the tagging of the primers, which is necessary for most next-generation sequencing techniques in order to analyse individual samples [44], can influence their reactivity in the PCR [45].

Due to these constraints, next generation sequencing is recommended to situations where little or no *a priori* knowledge is available. Alternatively, when information on the population level is sufficient for addressing a study's aims, a meta-sample can be analysed [46]. However, in-depth analysis will be limited to a few individual samples only (e.g. [20],[22]) due to the cost of this approach. Moreover, it is expensive to use separate tags for potentially hundreds of individual samples. Hence, for work which requires an individual-based analysis, primers can subsequently be designed that target specific taxa followed by mass screening of individuals, using multiplexing and fragment analysis to make the task more efficient [47].

While in next generation sequencing species are identified by comparing the obtained DNA with reference sequence information, in diagnostic PCR, plant identification is based on differences in amplicon size. Hence, it is vital for the current approach that the specific amplicon sizes are obtained with target DNA only, involving the need to carefully test the PCRs against a wide range of non-target taxa whose DNA might be also present in the samples [48]. Accordingly, recurrent checks of a subsample of amplicons via sequencing are advisable to confirm the identity of the target species. For the PCR protocols presented here the levels of cross-reactivity remained low as in only one case a size-specific PCR product was obtained with a non-target species, belonging to the same family. An application on other plant communities will require cross-reactivity testing with species that were not present in the current study.

Our PCR assays were successful in detecting as less as 100 template molecules per reaction. The sensitivity remained high even in the presence of excess non-target (wireworm) DNA, mimicking plant detection in complex mixtures of DNA, as it is the case for gut content-, faecal-, litter- or soil samples. Besides a high assay sensitivity, PCR products need to be short enough to track degraded DNA [10], like remains in decaying plant material as well as in the intestine and faeces of herbivores [23]. The current assays generate amplicons with less than 400 bp, thus maximizing the likelihood of detection of degraded DNA.

We already proved the capability of our approach to detect and identify DNA of ingested plants from whole-body extracts of wireworms for over three days post-feeding [31]. Here, we introduce two methods to increase the efficacy of diagnostic screenings. Firstly, primer pairs have been combined in multiplex PCRs to reduce the number of PCRs necessary [42]. Secondly, the application of family- primers allows a pre-selection of samples, which considerably reduces the number of samples that need to be analysed for different genera or species within this family.

Our molecular identification system could also be applied in forensic botany to routinely and correctly identify trace botanical evidence, where the absence of an accurate identification system currently remains the major obstacle [49]. For analysis of botanical trace evidence in criminal and civil cases plant species identification would be reduced to a set of PCRs in a routine analysis based on the PCR technique reported here. Tsai et al. [17] established a DNA database of local plants in Taiwan from sequences comprising the *trn*L intron and the *trn*L-F intergenic spacer, which could provide an additional basis for the development of new specific primers.

The analysis of leaf litter mixtures is another example where decaying plant material is difficult to assign to species [50]. Although badly needed - to our knowledge - currently no successful attempts of molecular litter identification exist. It is very difficult to estimate litter composition in natural ecosystems: Many species are mixed, and they are present in different stages of decay due to species dependent differences in rates of plant-litter decomposition [51]. This causes problems when attempting to sequence litter samples. The use of short diagnostic PCR products as markers enables the detection, even if only traces of DNA are left. It provides a simple and cheap means for sorting litter components into species, similar to the molecular identification of detritivorous macro-invertebrates from their faecal pellets [52].

In summary, the approach outlined here is applicable for the identification of otherwise unidentifiable plant(part)s, comprising roots, leaf litter, decaying or ingested plant material, and herbivore faeces. It offers a wide range of application and can be tailored towards the needs of future work following the protocols described here, contributing to a better understanding in what is going on “directly under our very noses”.

## Supporting Information

Supporting Information S1 PCR conditions to generate templates for sequencing.
(DOC)

Supporting Information S2 PCR conditions for initial primer testing.
(DOC)

Supporting Information S3 PCR conditions for template generation.
(DOC)

## References

[1] McNickle G, Cahill J, Deyholos M (2008). A PCR-based method for the identification of the roots of 10 co-occurring grassland species in mesocosm experiments.. Botany.

[2] Taggart J, Cahill J, McNickle G, Hall J (2011). Molecular identification of roots from a grassland community using size differences in fluorescently labelled PCR amplicons of three cpDNA regions.. Molecular Ecology Resources.

[3] Casper B, Jackson R (1997). Plant competition underground.. Annual Review of Ecology and Systematics.

[4] Johnson S, Murray P (2008). Root feeders and ecosystem perspective; Johnson S, Murray P, editors.

[5] Jackson R, Moore L, Hoffmann W, Pockman W, Linder C (1999). Ecosystem rooting depth determined with caves and DNA.. Proceedings of the National Academy of Sciences of the USA.

[6] Linder C, Moore L, Jackson R (2000). A universal molecular method for identifying underground plant parts to species.. Molecular Ecology.

[7] Valentini A, Pompanon F, Taberlet P (2009). DNA barcoding for ecologists.. Trends in Ecology & Evolution.

[8] Hebert P, Cywinska A, Ball S, deWaard J (2003). Biological identifications through DNA barcodes.. Proceedings of the Royal Society B: Biological Sciences.

[9] Chase M, Cowan R, Hollingsworth P, van den Berg C, Madriñán S (2007). A proposal for a standardised protocol to barcode all land plants.. Taxon.

[10] Taberlet P, Coissac E, Pompanon F, Gielly L, Miquel C (2007). Power and limitations of the chloroplast *trn*L (UAA) intron for plant DNA barcoding.. Nucleic Acids Research.

[11] Fazekas A, Burgess K, Kesanakurti P, Graham S, Newmaster S (2008). Multiple multilocus DNA barcodes from the plastid genome discriminate plant species equally well.. PLOSone.

[12] Hollingsworth M, Clark A, Forrest L, Richardson J, Pennington R (2009). Selecting barcoding loci for plants: evaluation of seven candidate loci with species-level sampling in three divergent groups of land plants.. Molecular Ecology Resources.

[13] Vijayan K, Tsou C (2010). DNA barcoding in plants: taxonomy in a new perspective.. Current Science.

[14] Taberlet P, Gielly L, Pautou G, Bouvet J (1991). Universal primers for amplification of three non-coding regions of chloroplast DNA.. Plant Molecular Biology.

[15] Borsch T, Hilu K, Quandt D, Wilde V, Neinhuis C (2003). Noncoding plastid trnT-trnF sequences reveal a well resolved phylogeny of basal angiosperms.. Journal of Evolutionary Biology.

[16] Ridgway K, Duck J, Young J (2003). Identification of roots from grass swards using PCR-RFLP and FFLP of the plastid trnL (UAA) intron.. BMC Ecology.

[17] Tsai L, Yu Y, Hsieh H, Wang J, Linacre A (2006). Species identification using sequences of the trnL intron and the trnL-trnF IGS of chloroplast genome among popular plants inTaiwan.. Forensic Science International.

[18] Spaniolas S, Bazakos C, Spano T, Zoghby C, Kalaitzis P (2010). The potential of plastid trnL (UAA) intron polymorphisms for the identification of the botanical origin of plant oils.. Food Chemistry.

[19] Pegard A, Miquel C, Valentini A, Coissac E, Bouvier F (2009). Universal DNA-Based methods for assessing the diet of grazing livestock and wildlife from feces.. Journal of Agriculture and Food Chemistry.

[20] Valentini A, Miquel C, Nawaz M, Bellemain E, Cossiac E (2009). New perspectives in diet analysis based on DNA barcoding and parallel pyrosequencing: the *trn*L approach.. Molecular Ecology Resources.

[21] Rayé G, Miquel C, Coissac E, Redjadj C, Loison A (2011). New insights on diet variability revealed by DNA barcoding and high-throughput pyrosequencing: chamois diet in autumn as a case study.. Ecological Research.

[22] Soininen E, Valentini A, Coissac E, Miquel C, Gielly L (2009). Analysing diet of small herbivores: the efficiency of DNA barcoding coupled with high-throughput pyrosequencing for deciphering the composition of complex plant mixtures.. Frontiers in Zoology.

[23] King R, Read D, Traugott M, Symondson W (2008). Molecular analysis of predation: a review of best practice for DNA-based approaches.. Molecular Ecology.

[24] Edwards M, Gibbs R (1994). Multiplex PCR: advantages, development, and applications.. Genome Research.

[25] Bakker F, Culham A, Gomez-Martinez R, Carvalho J, Compton J (2000). Patterns of nucleotide substitution in angiosperm cpDNA trnL (UAA) –trnF (GAA) regions.. Molecular Biology and Evolution.

[26] Mommer L, Wagemaker C, DeKroon H, Ouborg N (2008). Unravelling below-ground plant distributions: a real-time polymerase chain reaction method for quantifying species proportions in mixed root samples.. Molecular Ecology Resources.

[27] Lee E, Hwang I, Kim N, Lee K, Han M (2010). An assessment of the utility of universal and specific genetic markers for opium poppy identification.. Journal of Forensic Sciences.

[28] Traugott M, Schallhart N, Kaufmann R, Juen A (2008). The feeding ecology of elaterid larvae in Central European arable land: new perspectives based on naturally occurring stable isotopes.. Soil Biology & Biochemistry.

[29] Hill D (1987). Agricultural insect pests of temperate regions and their control.

[30] Hall T (1999). BioEdit: A user-friendly biological sequence alignment editor and analysis program for Windows.. Nucleic Acids Symposium Series.

[31] Staudacher K, Wallinger C, Schallhart N, Traugott M (2011). Detecting ingested plant DNA in soil-living insect larvae.. Soil Biology and Biochemistry.

[32] Moore L, Field C (2005). A technique for identifying the roots of different species in mixed samples using nuclear ribosomal DNA.. Journal of Vegetation Science.

[33] Pumarino L, Alomar O, Agusti N (2010). Development of specific ITS markers for plant DNA identification within herbivorous insects.. Bulletin of Entomological Research.

[34] Kesanakurti P, Fazekas A, Burgess K, Percy D, Newmaster S (2011). Spatial patterns of plant diversity below-ground as revealed by DNA barcoding.. Molecular Ecology.

[35] Hollingsworth P, Forrest L, Spouge J, Hajibabaei M, Group: CPW (2009). A DNA barcode for land plants.. PNAS.

[36] Bobowski B, Hole D, Wolf P, Bryant L (1999). Identification of roots of woody species using polymerase chain reaction (PCR) and restriction fragment length polymorphism (RFLP) analysis.. Molecular Ecology.

[37] Brunner I, Brodbeck S, Büchler U, Sperisen C (2001). Molecular identification of fine roots of trees from the Alps: reliable and fast DNA extraction and PCR–RFLP analyses of plastid DNA.. Molecular Ecology.

[38] Donini P, Elias M, Bougourd S, Koebner R (1997). AFLP fingerprinting reveals pattern differences between template DNA extracted from different plant organs.. Genome.

[39] Jurado-Rivera J, Vogler A, Reid C, Petitpierre E, Gómez-Zurita J (2009). DNA barcoding insect–host plant associations.. Proceedings of the Royal Society B.

[40] Navarro S, Jurado-Rivera J, Gomez-Zurita J, Lyal C, Vogler A (2010). DNA profiling of host-herbivore interactions in tropical forests.. Ecological Entomology.

[41] Schnell B, Fraser M, Willerslev E, Gilbert M (2010). Characterisation of insect and plant origins using DNA extracted from small volumes of bee honey.. Arthropod-Plant Interactions.

[42] Harper G, King R, Harwood J, Glen D, Bruford M (2005). Rapid screening of invertebrate predators for multiple prey DNA targets.. Molecular Ecology.

[43] Traugott M, Bell J, Broad G, Powell W, van Veen F (2008). Endoparasitism in cereal aphids: molecular analysis of a whole parasitoid community.. Molecular Ecology.

[44] Glenn T (2011). Field guide to next-generation DNAsequencers.. Molecular Ecology Resources.

[45] Pompanon F, Deagle B, Symondson W, Brown D, Jarman S (in press). Who is eating what: diet assessment using Next Generation Sequencing.. Molecular Ecology.

[46] Deagle B, Kirkwood R, Jarman S (2009). Analysis of Australian fur seal diet by pyrosequencing prey DNA in faeces.. Molecular Ecology.

[47] King R, Moreno-Ripoll R, Agusti N, Simon P, Shayler S (2010). Multiplex reactions for the molecular detection of predation on pest and nonpest invertebrates in agroecosystems.. Molecular Ecology Resources.

[48] Admassu B, Juen A, Traugott M (2006). Earthworm primers for DNA-based gut content analysis and their cross-reactivity in a multi-species system.. Soil Biology and Biochemistry.

[49] Ferri G, Alù M, Corradini B, Beduschi G (2009). Forensic botany: species identification of botanical trace evidence using a multigene barcoding approach.. International Journal of Legal Medicine.

[50] Gartner T, Cardon Z (2004). Decomposition dynamics in mixed-species leaf litter.. Oikos.

[51] Cornelissen J, Perez-Harguindeguy N, Diaz S, Grime J, Marzano B (1999). Leaf structure and defence control litter decomposition rate across species and life forms in regional floras on two continents.. New Phytologist.

[52] Seeber J, Rief A, Seeber G, Meyer E, Traugott M (2010). Molecular identification of detritivorous soil invertebrates from their faecal pellets.. Soil Biology and Biochemistry.

