# Supplementary material for: Rapid Plant Identification Using Species- and Group-Specific Primers Targeting Chloroplast DNA
Source: PLoS One. 2012 Jan 12;7(1):e29473. doi: 10.1371/journal.pone.0029473 (PMC3257244; doi:10.1371/journal.pone.0029473)
Supplement: Supporting Information S1 — PCR conditions to generate templates for sequencing. (DOC) [file pone.0029473.s001.doc]

The 15 µL PCR mix contained 0.2 mM dNTPs (Ares Bioscience, Köln, Germany), 1 μM of each primer, 1× Reaction Buffer, 3 mM MgCl_2_, 0.56 U BioTherm^TM^ Taq DNA Polymerase (Ares Bioscience) and 1 µL DNA extract. The thermocycling program (executed on a Mastercycler Gradient, Eppendorf, Hamburg, Germany) was 94 °C for 2 min, 35 cycles of 94 °C for 20 s, 50–57 °C (depending on primer combination and plant species) for 60 s and 70 °C for 60 s, and a final elongation of 2 min at 72 °C. PCR products were visualized in agarose gels stained with GelRed^TM^ (Biotium, Hayward, USA). Of each PCR product 5 µL were purified with 0.5 U SAP (Shrimp Alkaline Phosphatase, Promega, Madison, USA) and 1 U Exonuclease 1 (New England Biolabs, Ipswich, US). Cycle Sequencing PCR was done with BigDye® Terminator v3.1 Cycle Sequencing Kit (Applied Biosystems, Foster City, USA) following the manufacturers protocol. PCR products were pelletized and cleaned using isopropanol and ethanol. Sequences were generated using a 3130 Genetic Analyser (Applied Biosystems).
