# Supplementary material for: Rapid Plant Identification Using Species- and Group-Specific Primers Targeting Chloroplast DNA
Source: PLoS One. 2012 Jan 12;7(1):e29473. doi: 10.1371/journal.pone.0029473 (PMC3257244; doi:10.1371/journal.pone.0029473)
Supplement: Supporting Information S2 — PCR conditions for initial primer testing. (DOC) [file pone.0029473.s002.doc]

PCRs were performed in 10 µL reactions containing 0.2mM dNTPs (Ares Bioscience), 1 μM of each primer, 1× Reaction Buffer, 3 mM MgCl_2_, 0.5 µg bovine serum albumin (BSA), 0.375 U BioTherm^TM^ *Taq* DNA Polymerase (Ares Bioscience), and 1.5 µL DNA extract. The thermocycling program varied slightly between the primer combinations: 94 °C for 2 min, 35/40 cycles of 94 °C for 20  s, 56–60 °C for 30/60 s and 70/72 °C for 45/60 s and finally 70/72 °C for 2  min. PCR products were visualized in agarose gels stained with GelRed (Biotium).
