# Supplementary material for: Rapid Plant Identification Using Species- and Group-Specific Primers Targeting Chloroplast DNA
Source: PLoS One. 2012 Jan 12;7(1):e29473. doi: 10.1371/journal.pone.0029473 (PMC3257244; doi:10.1371/journal.pone.0029473)
Supplement: Supporting Information S3 — PCR conditions for template generation. (DOC) [file pone.0029473.s003.doc]

Each 15 µl PCR contained 2.25 µL of DNA extract, 0.563 U BioTherm^TM^ *Taq* DNA Polymerase (Ares Bioscience), 1× Reaction Buffer, 3 mM MgCl_2_, 0.2 mM dNTPs Ares Bioscience), 1 µM of each primer, 7.5 µg BSA and 5 µL of PCR-grade RNase-free water (Qiagen). 94 °C for 2 min, 35 cycles of 94 °C for 20 s, 50–57 °C (depending on primer combination and plant species) for 60 s and 70 °C for 60 s, and a final elongation of 2 min at 72 °C. PCRs were repeated to gain 45 µL of PCR product per species in total. Out of this, 40 µL were purified with QIAquick PCR Purification Kit (Qiagen), and finally eluted with 50 µL of 1×TE buffer and subjected to DNA concentration measurement.
